# Method for the detection of powdery mildew in tomato from electrical signalling

**DOI:** 10.1016/j.mex.2026.103838

**Published:** 2026-02-20

**Authors:** Slavica Matic, Giorgio Masoero, Pier Paolo Capra, Andrea Sosso

**Affiliations:** a Institute for Sustainable Plant Protection (IPSP), National Research Council of Italy (CNR), Strada delle Cacce 73, Torino 10135, Italy; b Department of Agricultural, Food and Forest Sciences (SAAF), University of Palermo, Viale delle Scienze, Palermo 90128, Italy; c Accademia di Agricoltura di Torino, Palazzo Corbetta Bellini di Lessolo, Via Andrea Doria 10, Torino 10100, Italy; d Department of Agricultural, Forest and Food Sciences (DISAFA), University of Turin, Grugliasco, Italy; e Istituto Nazionale di Ricerca Metrologica (INRiM), Strada delle Cacce 91, Torino 10135, Italy

**Keywords:** Tomato, Powdery mildew, Electrical potential, Foliar pH, Early diagnosis, PLS-D

## Abstract

Plants are known to generate various types of electrical signals, which have been observed ever since Darwin’s times. We studied the electrical signals acquired in tomato plants infected with the fungal pathogen *Oidium neolycopersici (On)*, the causative agent of powdery mildew, and applied statistical analyses to detect the differences in electrical responses between healthy and infected plants, as reported in [1].

• The underlying mechanism in the generation and transmission of electrical signals is not fully understood, yet it’s generally accepted that they can be classified according to functional properties. Action potentials (APs) and slow wave potentials, in particular, are elicited by biotic and abiotic stimuli, thus are interesting as a hallmark of plant health status.
• To analyse the application of these potentials in plant disease detection, voltages from electrodes inserted in plants were acquired periodically by a scanning multimeter and recorded under control of a dedicated custom Python program running on a Raspberry Pi board.
• Here we describe the design of the experiment and analyse in some detail the solutions adopted for specific issues found in the measurements, such as electrode’s material and placement; immunity to electromagnetic noise; data logging over long periods of time with intermediate monitoring of results.

## Graphical abstract

**Figure:**
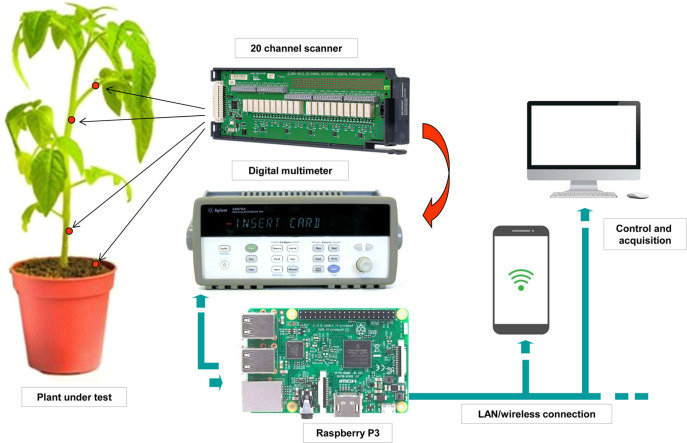

**Specifications table**

**Table:** 

**Subject area**	*Plant Pathology*
**More specific subject area**	*Precision Agriculture, Non-destructive Diagnosis*
**Name of your method**	*Powdery mildew detection in tomato from electrical signalling*
**Name and reference of original method**	*n/a*
**Resource availability**	Repository name: Mendeley Data Data identification number: 10.17632/yr8zhsc6mh.1 Direct URL to data: https://data.mendeley.com/datasets/yr8zhsc6mh/1

## Background

There is an increasing interest in electrophysiological plant responses to abiotic stress such as cold, mechanical injury, and water scarcity as well as to biotic stressors like pest injury. Yet, monitoring of electrical signals in plants affected by pathogens is still in its early stages, with studies generally over observation periods ranging from seconds to hours, focused on medium frequency ranges, and no long-term monitoring of electrical potential in the course of infection.

Powdery mildew was considered the proper choice for our tests: a prominent tomato disease; difficult to monitor due to subtle symptoms; spread over distances up to kilometers; easily transported by personnel and machinery; requires multiple treatments throughout the life cycle; dramatic reduction in yield and product quality of infected plants; innovative diagnostic methods for disease detection before the appearance of symptoms are sought.

Additionally, only few non-destructive techniques, as potential sensing, have been developed, for the detection of tomato powdery mildew, like: thermal/light imaging, RGB imaging, machine and deep learning systems [[2], [3], [4], [5], [6], [7]]. In contrast to these techniques, which rely on temperature, colour and spectral reflectance data, electrical signalling, another non-destructive technique, is based on electrical data. Notably, only one study has focused on electrical signal measurements in tomato plants affected by powdery mildew [8].

Overall, electrical signals provide a solution in between chemical and non-contact analyses, offering analysis at a microscale level similar to mass spectrometry–based techniques and providing the simplicity and velocity similar to imaging methods. Another objective was to evaluate whether the electrical potential was conditioned by the different type of substrate, water and peat, in which tomato plants were grown. Finally, a correlation model connecting the electrical potential in plants to the disease symptoms is presented [[9], [10], [11]].

## Method details

Among the different electrical potentials generated by plants reported in literature, the long-term APs was selected as a possible indicator of a plant illness condition in the experiment.

To that aim, electrodes were inserted in tomato plants infected with powdery mildew and the electrical signals recorded for 15 days during infection. At the same time, signals from electrodes in non inoculated tomato plants were observed as healthy controls.

To grow plants used in the experiment, tomato seeds were germinated in plug trays, and after 2 weeks the seedlings were transplanted into 1-L pots containing a peat-based mixture substrate (6:2:1 by volume of peat/coconut granules/volcanic pumice;) and a NPK+microelements slow release fertilizer (Vigorplant, Fombio (LO), Italy) and 1-L flasks containing unsupplemented tap water; 6 plants per each substrate. The MB1 isolate of the phytopathogenic fungus On [12] was maintained on tomato cv. Marmande under greenhouse conditions (23 °C day, 19 °C night, and 70 % relative humidity). Three of the six 1-month old plants were artificially inoculated by On as described by [12] while three remaining non-inoculated plants were used as the healthy control plants. Inoculation was performed by spraying of two primary leaflets of three randomly selected leaves with 10 μL of conidial suspension (5 × 10^−4^ spores/mL), prepared by detaching spores from heavily infected sporulating leaves. Following inoculation, plants were cultivated for an additional 22 days and visually checked for powdery mildew disease progress. The On symptoms were assessed by visual observation at 8, 15, and 22 days post-inoculation (dpi), evaluating the disease index (DI) through the percentage of the affected leaf area. All DI were evaluated simultaneously, observing the same leaflets until 22 dpi to ensure full development of symptoms. To confirm the infection by On, the ITS sequence was amplified through PCR using ITS4 and ITS5 primers on DNA extracted from symptomatic leaves of tomato plants. The PCR amplicons (595–596 bp) were sequenced by the Sanger method and confirmed as On. The evaluated DI was then linearly regressed on the dpi to obtain daily a disease rate (DR) for each plant. The experiment was repeated twice and the results mediated between the two repetitions.

**Fig. 1:**
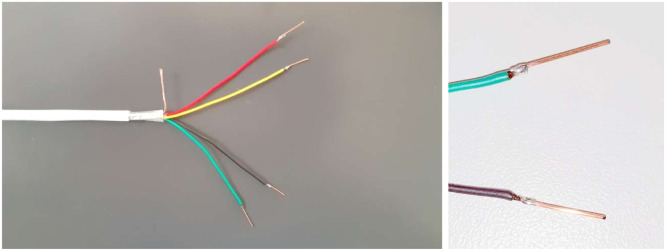
Multi-core cable (left) for connecting pure-gold electrodes (right).

Electrical measurements were performed by means of pure gold custom-made electrodes with 0.1 mm diameter and 10 mm length, inserted into the plants’ stems down to a depth of 3 mm, in order to reach the conducting bundles, one day before to the electrical signal recording, to acclimate. The data acquisition unit was realized with an Agilent 34970A 6½-digit [13] Digital Multimeter (DMM) equipped with a 34901A multiplexer module. This configuration was selected for its versatility, accuracy, and ease of integration: The DMM can accommodate up to three internal scanner modules, offering scalability depending on the number of channels required. Each scanner module provides 20 two-wire measurement channels (plus 2 for control/reference), enabling voltage, current, and resistance measurements. The modules are based on electromechanical relays, which ensure high accuracy and low offset currents, suited for slower switching speed as required in this case. In the setup (shown in Graphical abstract), screw-terminal connectors are used to contact the measurement wires. Each plant is connected at multiple points using shielded multi-core cables terminated with pure gold electrodes (see Fig. 1), selected for their resistance to oxidation. For voltage measurements, one channel with separate ground is assigned to monitor every electrode in the plant, with shield connected to ground reference potential, to minimize noise and interferences.

**Fig. 2:**
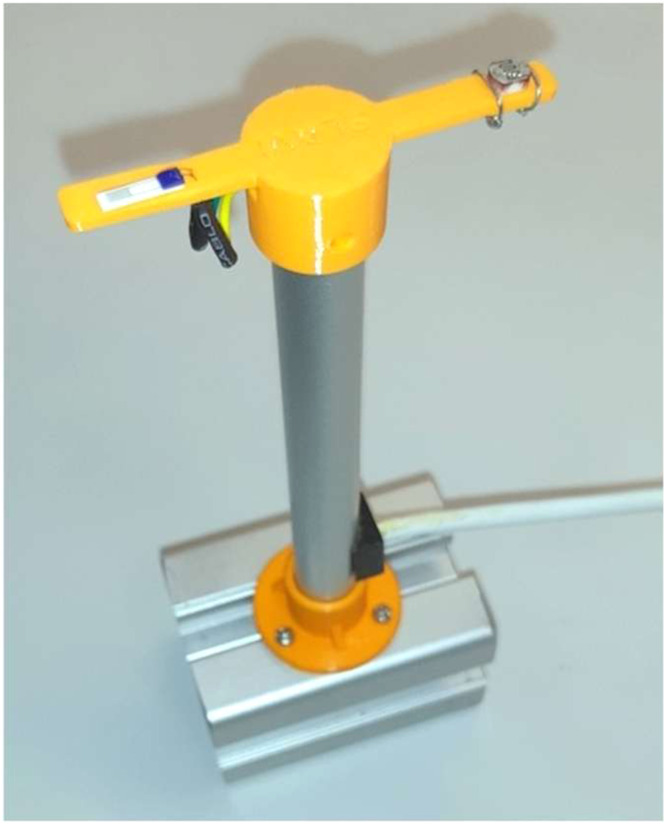
Photograph of the stand supporting the thermometer (left) and light sensor (right).

Environmental parameters, temperature and light intensity are monitored using two dedicated channels for sensors placed on a custom designed stand in close proximity to the plants (see Fig. 2) Temperature is measured using a platinum resistance thermometer (Pt100), while light intensity is monitored via a photoresistor.

The instrument was set to auto-range for maximum flexibility in handling potential variations, with 200 Power-Line-Cycles integration time to optimize noise reduction, avoiding the need of a Faraday cage in detecting signals at the mV level. Additionally, the internal prefilter was set to low pass with 3 Hz cut-off frequency, to observe plant electrical activity at low frequencies, avoiding electrical disturbances and unwanted noise.

Remote control of the DMM unit is available via serial interface (RS-232) and IEEE-488 (GPIB). In the present configuration, a USB-to-serial converter is used to connect the DMM serial interface to a Raspberry Pi single-board computer with Debian GNU/Linux 12 (bookworm) [14] operating system that handles the whole measurement procedure including data acquisition and storage on both the internal memory and a USB removable memory key. It can be accessed remotely via WiFi through the internal server with secure SSH protocol, that allow to take full control of all computer functions, including encrypted fast transfer of measurement data. Alternatively, the data can be transferred for analysis by removing the USB storage key. Key removal, automatically stops measurements, that are restarted at reinsertion of the USB key. Furthermore, the measurement program is auto-started at reboot of the operating system, to guarantee recovery in case of failure, due, e.g., to a mains outage, and minimal losses of experimental data.

The software to manage the whole process integrates different languages. First, a simple bash shell script that runs automatically at system boot, in charge of starting the measurement program and restart it indefinitely if program terminates. The actual measurement program executes a Python code that takes care of the initialization and configuration of the DMM, to manage the scanning sequence over selected channels for voltage (generally all 20), the thermometer and the light sensor, reading every value. To summarize, the object-oriented code to manage measurements is organized on a hierarchical structure:

• At lowest level the SeriaLine base communication class sets up the serial interface for I/O operation and defines the timeout (Supplementary Materials, Box S1).
• A generic serial-connected device is controlled by the Device_IntFace class where method to write commands to the device and query data are defined. The class is built from the SerLine class that is instantiated in the initialization method (Supplementary Materials, Box S2).
• The Control_34970A class is a specialized code to operate with the DMM model in the experiment. It inherits from Device_IntFace and implements the commands to control the multimeter and close the communications properly (Supplementary Materials, Box S3).
• Finally, the code that implements the measurement procedure and sends configuration commands to the DMM first, then starts the loop that continuously reads the electrodes voltages and sensors resistance values, saving to a Comma-Separated-Values file the results (Supplementary Materials, Box S4).

## Method validation

The differences in electrical potential observed during the experiment between infected and healthy plants were validated against the visual assessment of powdery mildew symptoms. The clear presence of symptoms on infected plants and the absence of symptoms on healthy plants from 5 to 15 dpi confirmed the difference in electrical potential between the two groups. This validation was possible from 5 to 15 days of electrical potential measurement, as no symptoms were seen in the earlier period. To confirm that the pathogen was present in the first four days of measurement, observations of fungal conidia on infected leaves were made every 6 h during the 4 dpi and the presence of conidia and progressive growth of fungal mycelium were confirmed during this period. Importantly, the ability of electrical monitoring to detect infection as early as 1.9 dpi compared to symptom observation (5 dpi) indicates that bioelectrical signals not only mirror the disease index but also anticipate visible symptoms, validating this approach as a complementary and potentially earlier marker of plant health status.

## Limitations

Electrical signals in plants originate from chemical processes within their tissues, and their measurement is influenced by various electrochemical factors. Plants exhibit four distinct types of these signals, characterized by different speeds and frequencies. To improve signal stability, it's important to reduce background noise. This can be achieved using shielded cables, digital signal processing, and differential measurement circuits. By filtering the input, one selects the relevant signals and minimizes noise, but some interesting information may be lost.

Due to the experiment's multi-day duration, the large volume of recorded data could complicate statistical analysis. While reducing the data acquisition frequency can simplify this, it increases the risk of missing rapid signal changes.

Moving from controlled laboratory settings to real-world field conditions is advisable, yet a significant challenge for electrical monitoring. The main difficulty is ensuring long-term electrode stability when faced with environmental factors like wind, rain, and plant growth. The choice of electrode material is crucial as it can directly impact the accuracy of results. To improve performance in the field, electrodes should be hydrophobic and highly corrosion-resistant. While gold is a common choice due to its resistance to contamination, other options exist. Platinum and stainless steel are excellent alternatives because, as gold, they are not prone to oxidation. Silver/Silver Chloride (Ag/AgCl) electrodes are also widely used because they are non-polarizable and biocompatible. Materials like gold-coated or carbon-based materials with self-cleaning abilities are ideal for withstanding high humidity and rainfall. Proper voltage measurements depend on maintaining stable contact between the electrode and the plant. Clip-on flexible electrodes with adaptable mounting and strain-relief properties help to maintain stable contact even as plants move due to growth and wind.

Ultimately, more durable field trials are needed to assess the long-term robustness of electrodes, confirm calibration procedures, and ensure that these systems can be successfully scaled for large-scale agricultural applications. For continuous monitoring, instruments with wireless sensor nodes are an ideal solution. These nodes, equipped with solar energy elements and low-power microcontrollers, can record and transmit real-time data using technologies like Bluetooth, NB-IoT, and LoRa. Integrating these sensors into IoT-based platforms and combining electrical recordings with other sensing methods—like imaging or plant performance sensors—could help create predictive models for diseases and early warning systems for pest management.

## Ethics statements

The authors have read and followed the ethical requirements for publication in Data in Brief and confirm that the current work does not involve human subjects, animal experiments, or any data collected from social media platforms.

## CRediT author statement

**Slavica Matic**: Conceptualization, Methodology, Investigation, Resources, Data Curation, Writing - Original Draft, Writing - Review & Editing, Supervision. **Giorgio Masoero**: Conceptualization, Formal analysis. **Pier Paolo Capra**: Investigation, Resources. **Andrea Sosso**: Conceptualization, Methodology, Software, Investigation, Writing - Original Draft, Writing - Review & Editing, Supervision.

## Declaration of competing interest

The authors declare that they have no known competing financial interests or personal relationships that could have appeared to influence the work reported in this paper.

## Data Availability

Published: Data in Brief

## Appendix Supplementary materials

Image, application 1
