# Supplementary material for: Method for the detection of powdery mildew in tomato from electrical signalling
Source: MethodsX. 2026 Feb 20;16:103838. doi: 10.1016/j.mex.2026.103838 (PMC12950449; doi:10.1016/j.mex.2026.103838)
Supplement: Supplementary file 1 [file mmc1.docx]

**Supplementary material *and/or* additional information**

import serial.tools.list_ports

import serial

import time

class SeriaLine:

def __init__(self, com_port=0, baudrate_var=115200):

com_device = f'/dev/ttyS{com_port}'

# comport for PC developm. Comment out for Raspberry

com_device = f'/dev/ttyUSB{com_port}'

com_devices_list = [comport.device for comport in                             serial.tools.list_ports.comports()]

if com_device not in com_devices_list:

print("COM port was not found")

print("Please ensure that USB is connected")

print(f"Please check COM port Number. Currently it is                                                               {com_port} ")

print(f'Found COM ports:{com_devices_list}')

else:

self.serline = serial.Serial(

port=com_device,

baudrate=baudrate_var,

xonxoff=False,

timeout=1,

write_timeout=0.01

)

if not self.serline.isOpen():

self.serline.open()

# tmp = self.serline.isOpen()

# print("is open:", tmp)

# return_value = self.get_status()

def timeout(self,time):

# set timeout in seconds

self.serline.timeout(time)

def close(self):

self.serline.close()

self.serline = None

Box S1: Class SeriaLine manages low-level serial communications

class Device_IntFace:

def __init__(self, *args):

self.serialconn = SeriaLine(*args)

### Test communication

cmmd = '*IDN?'

read_back = self.query(cmmd)

print(f"\nConnected to: {read_back}")

def write(self, cmd_srt):

time.sleep( 0.2 )

self.cmmd = f'{cmd_srt}\r\n'

self.serialconn.serline.write(self.cmmd.encode())

def query(self, cmd_srt):

self.write(cmd_srt)

time.sleep( 0.2 )

self.answ = self.serialconn.serline.readline()

# Check if not regular \n (ascii 10) terminat

if len(self.answ) == 0 or self.answ[-1] != 10:

print("Serial communication error",self.answ)

raise SystemExit(1)

# ''.join( ch for ch in self.answ if ch.isalnum() )

return self.answ.decode().strip()

def timeout(self,time):

self.serialconn.timeout(time)

def close(self):

self.serialconn.close()

self.serialconn = None

Box S2: Class Device_IntFace: the high-level code to configure, send commands and receive data,
to be used with a generic instrument

class Control_34970A(Device_IntFace):

"""

Args:

param1 (Optional[int]): Serial port number

param2 (Optional[int]): Serial baud rate

"""

def __init__(self, *args):

super().__init__(*args)

def get_34970A_date(self):

read_back = self.query( 'SYST:DATE?' )

print(f"SYST:DATE? {read_back}")

return read_back

def get_34970A_time(self, hh=None, mm=None, ss=None, check_val=0):

try:

tstsm = hh + mm + ss

except:

hh = self.t_now.tm_hour

mm = self.t_now.tm_min

ss = self.t_now.tm_sec

hh = range_check( hh, 0, 23, "hours")

mm = range_check( mm, 0, 59, "minutes")

ss = range_check( ss, 0, 59, "seconds")

ss = round(ss,3)

check_val = range_check(check_val, 0, 1, "check_back bool val" )

cmmd = f'SYST:TIME {str(hh).zfill(2)},{str(mm).zfill(2)},{str(ss).zfill(6)}'

self.write( cmmd )

if check_val == 1:

read_back = self.query( 'SYST:TIME?' )

print(f"SYST:TIME? {read_back}")

return read_back

def conf_reading_time(self, on_off, check_val=1):

'''

configuring reading time: on_off = 0/off, 1/on

'''

cmd_list = ["OFF", "ON", "Unknown"]

on_off = range_check(on_off,0,1,"ON/OFF state")

check_val = range_check(check_val, 0, 1, "check_back bool val")

cmmd = 'FORM:READ:TIME'

self.send( cmmd + cmd_list[on_off] )

if check_val == 1:

read_back = int(self.query( cmmd+'?' ))

print(f"FORM:READ:TIME {cmd_list[read_back]}")

return read_back

def get_sys_time_scan(self, show_val=0):

show_val = range_check(show_val, 0, 1, "show bool val")

txt = 'SYST:TIME:SCAN?'

self.ser.write(txt.encode())

read_back = self.ser.readline().decode()

if show_val ==1:

print(f"{read_back}")

return read_bac

Box S3: Class Control_34970A, a child of Device_IntFace, implements specific commands

to operate with the Agilent 34970A DMM.

from datetime import datetime

import os.path

import csv

import time

''' Set Konstants '''

USBmntPath = "/run/media/andrea/AgriDisk" # development stage

#USBmntPath = "/media/agrinext/AgriDisk" # Raspberry mount

nplc = 200

numberChns = 20

chnDelay = 8 # (auto) # delay in secs, between relay closure and meas

scanWait = 20 # delay in secs, between end and new scan

scanPeriod = numberChns * (chnDelay-1) + scanWait

runTime = 1800 # total run time in secs

numberScans = int( runTime/scanPeriod ) # num of scans for runTime

''' GPIB interface '''

# os.add_dll_directory('C:\\Program Files\\Keysight\\IO Libraries Suite\\bin')

# import pyvisa

#

# '''

# Initialize the 34970A/72A and drivers

# load visa lib and open connection

# '''

# rm = pyvisa.ResourceManager('ktvisa32')

# IO_34970A = rm.open_resource('GPIB1::9::INSTR')

''' Serial interface '''

from Control_34970A import Control_34970A

IO_34970A = Control_34970A()

''' Measurement config '''

IO_34970A.timeout = 4

IO_34970A.write("*CLS")

IO_34970A.write("*RST")

''' Print Inserted cards

print( "\n", " * Test on board cards" )

scanlist = "(@"

for ch in range(1,4):

card_status = IO_34970A.query(f"SYST:CTYPE? {ch}00")

if card_status[-5:] != "0,0,0":

scanlist += f"{ch}01:{ch*100+int(numberChns/ch)}," ### !!! ###

scanlist = scanlist[:-1] + ")"

# scanlist does not have to include

# all configured channels

print("\n Scanlist:", scanlist, " Number of scans:", numberScans, "\n" )

''' setup channels configuration

IO_34970A.write( f"CONF:VOLT:DC AUTO,{scanlist}")

IO_34970A.write( f"SENS:VOLT:DC:NPLC {nplc}")

if "201" in scanlist:

""" If you select autoranging (by specifying "AUTO" or "DEF"), an

error will be generated if you specify a discrete value for the

<resolution> parameter. When autoranging is combined with a

discrete resolution, the instrument cannot accurately resolve the

integration time. If your application requires autoranging, be sure

to specify "AUTO" for the <resolution> parameter, or omit parameter

"""

IO_34970A.write( "CONF:RES AUTO,(@209)")

IO_34970A.write( "CONF:RES 10E+3,(@210)")

Box S4a: Code for measurement procedure with definition of constant values and DMM
configuration for measurement of electrodes voltage and sensors resistance

# setup scan list

IO_34970A.write( f"ROUTE:SCAN {scanlist}" )

numberChannels = int( IO_34970A.query("ROUTE:SCAN:SIZE?") )

IO_34970A.write( "FORMAT:READING:CHAN ON" )

IO_34970A.write( "FORMAT:READING:TIME ON" )

#IO_34970A.write( "FORMAT:READING:TIME:TYPE ABS" )

# channel delay

IO_34970A.write( "ROUT:CHAN:DELAY:AUTO ON" )

# setup when scanning starts and interval rate

IO_34970A.write( "TRIG:COUNT " + str( numberScans ))

IO_34970A.write( "TRIG:SOUR TIMER" )

IO_34970A.write( "TRIG:TIMER " + str( scanPeriod ))

while True:

''' Initiate Scan '''

# start the scan and retrieve the scan time

print ("\n Scan start time:", IO_34970A.query( "INIT; :SYSTEM:TIME:SCAN?"),"\n")

startMeas_time = time_34970A()

try: startMeas_time

except NameError: startMeas_time = datetime.now()

''' The data points are read: data, time, channel

'''

data_matrx = [ ['']*3*numberChannels for ns in range( numberScans ) ]

for scan in range( numberScans):

for chan in range( numberChannels):

# wait until there is a data available

points = 0

while ( points==0 ):

try:

points = int( IO_34970A.query( "DATA:POINTS?" ))

except ValueError:

points = 0

# read

data_rdng = IO_34970A.query( "DATA:REMOVE? 1" )

data_rdng = data_rdng.rstrip() # strip trailing /n

data_matrx[ scan ][ chan*3:chan*3+3 ] = reversed( data_rdng.split(",") )

data_matrx[ scan ][ chan*3+1 ] = f"{(time_34970A() -                       startMeas_time).seconds:07d}"

print( scan, chan, data_matrx[ scan ][ chan*3:chan*3+3 ] )

'''Save to CSV file

timeSave = startMeas_time.strftime("%y-%m-%d--%H%M%S")

with open( "Agln34970A_data_" + timeSave + ".csv", 'w', newline='' ) as csvfile:

filewrite = csv.writer( csvfile, dialect='excel-tab' )

for row in data_matrx:

filewrite.writerow(row)

while not os.path.ismount(USBmntPath):

print("... waiting for USB stick ready")

time.sleep(5)

time.sleep(5)

with open( USBmntPath + "/Agln34970A_data_" + timeSave + ".csv", 'w', newline='' ) as csvfile:

filewrite = csv.writer( csvfile, dialect='excel-tab' )

for row in data_matrx:

filewrite.writerow(row)

'''___ Close connection

IO_34970A.close()

# GPIB

# rm.close()

print('close instrument connection')

Box S4b: measurement loop running a scan to measure all channels sequentially,then save
results to a comma-separated-values file and repeat indefinitely
